# Germline testing, access to genetic counseling, and treatment implications in advanced breast cancer

**DOI:** 10.1097/MS9.0000000000004945

**Published:** 2026-04-08

**Authors:** Hafiza Qurat Ul Ain, Wajeeha Imam, Muhammad Junaid Tahir, Khabab Abbasher Hussien Mohamed Ahmed

**Affiliations:** a CMH, Multan, Pakistan; b Shaukat Khanum Memorial Hospital and Research Centre, Lahore, Pakistan; c Faculty of Medicine, University of Khartoum, Khartoum, Sudan

**Keywords:** breast cancer, genetic counseling, germline testing, palliative care

## Abstract

Metastatic breast cancer refers to the spread of cancer cells to distant parts of the body. Metastatic cells spread via the lymphatics or vascular system to form metastatic lesions, also called secondary tumors. Most cases are associated with mutations in the BRCA1 and BRCA2 genes. These defective genes pass from generation to generation. Germline testing helps in screening patients with a positive family history, thus aiding in protection from metastasis and helping in targeted therapy. The physician should offer genetic counseling to patients with a positive history of breast cancer. Treatment strategies include chemotherapy, hormonal therapy, radiotherapy, and immunotherapy. Surgical options include breast conservation surgeries and mastectomy, which is followed by breast reconstruction surgery. Palliative therapy includes control of symptoms and pain management. Supportive services such as counseling, support groups, and integrative therapy could assist patients and their families in overcoming emotional, social and spiritual needs. Besides treatment, clinical trials could enhance cancer research and lead to effective treatment.

Stage IV breast cancer, also known as advanced metastatic breast cancer, is a severe condition involving the spread of cancer cells to distant areas of the body beyond the breast tissue and adjacent lymph nodes. Metastatic cells involve the vascular or lymphatic system in metastatic breast cancer, enabling the spread to bones, liver, lungs, or brain. Metastases or metastatic lesions are the terms used to describe these secondary tumors^[^1^]^. It is imperative to take into consideration the fact that metastatic breast cancer is incredibly heterogeneous, and considerably differs in terms of site, metastasis, as well as characteristics of cancer cells (depending upon hormone receptor status i.e., HER 2 status)^[^2^]^.

Although advanced breast cancer is morbid, several approaches can help in the pre-determination of the risk, aiding in the protection from the devastating consequences of metastasis. DNA analysis to find particular genetic variations responsible for breast cancer is the process used in germline testing for advanced breast cancer^[^3^]^. Germline mutations are genetic variations that can be inherited by succeeding generations through germ cells, such as oocytes or sperm cells. Germline testing usually has a role in evaluating the risk of breast cancer with a positive family history and could be helpful in metastatic breast cancer to determine the eligibility for clinical trials and targeted therapy^[^4^]^. About 5–10% of women diagnosed with breast cancer have a pathogenic variant (PV) in hereditary cancer susceptibility of genes, having specific indications in managing these patients^[^1^]^.

Germline testing had been used previously to screen and reduce the risk of breast cancer. However, recently, genetic testing has been proved to be beneficial in diagnoses before the metastasis. With oral poly-ADP-ribose polymerase inhibitors, germline genetic data have important systemic therapeutic implications that can meaningfully enhance outcomes in breast cancer patients^[^5^]^.

The developments in systemic therapy require a paradigm shift for eligibility of germline investigations and changes in current testing models to satisfy the growing demand for germline testing. Despite the advantages of genetic testing, many eligible breast cancer patients remained uninvestigated attributing to the requirements of the National Comprehensive Cancer Network (NCCN). The American Society of Breast Surgeons had advocated germline genetic testing for the identification of BRCA1 and BRCA2 PVs for all breast cancer patients in 2019. Desai *et al* had outlined the shortcomings of BRCA1 and BRCA2 genetic testing recommendations and suggested a hybrid strategy to investigate all women diagnosed with breast cancer by age 65 and use NCCN criteria for older patients^[^2^]^. The optimal criteria of genetic testing should be sensitive enough to determine all the carriers of PV in a population and reduce the chances of indiscrimination for candidates with low incidence of a PV, and a negative result could cause severe distress.

The physician should offer genetic counseling to patients with a positive family history of breast cancer. Genetic counseling could help the patient and the family in informed decisions for genetic testing. The key points of genetic counseling are risk assessment, genetic testing and interpretation of test results, emotional support, education, and family communication^[^6^]^. The risk assessment for breast cancer is the evaluation of predisposing factors, such as family history, delayed puberty, menarche, and marriage age, lactation failure, late menopause, use of hormone replacement therapy, contraceptives, alcohol, smoking, lack of physical activity, and environmental intoxicants^[^7^]^. The genetic counselor should thoroughly investigate the medical history of patient and family, including any instances of breast cancer and other associated diseases. This evaluation aids in finding patterns pointing toward an inherited propensity for breast cancer.

Most inherited cases of breast cancer are associated with mutations in the BRCA1 and BRCA2 genes and are involved in the repair of damaged cells and the maintenance of normal cell growth. The inheritance of mutant genes from generation to generation could alter functionality and increase the risk of breast and ovarian cancer^[^6^]^. Protein-truncating mutations in the ATM, CHEK2, and PALB2 genes are also associated with an increased risk of breast cancer^[^5^]^. The genetic counselor should suggest genetic testing to find particular genetic mutations, which often entails blood or saliva sampling for laboratory analysis. The genetic counselor aids in the interpretation of the genetic testing, risk of acquiring breast cancer by the patient and the family, and also assists in decisions, responds to inquiries, and offers available resources. Genetic counselors assist patients in navigating family conversations regarding genetic testing and risk and encourage family discussions about the possible inherited danger and advice communication techniques.

A rise in the demand for counseling and testing is anticipated in the near future, attributing to clinical implications of the discovery of harmful mutations in BRCA, BRCA2, and other genes. Over 5 years, there is an expected increase in the workload to double (44%) or more than 100% (33%) for the doctors in terms of genetic counseling. All adequately trained professions, including clinical geneticists, genetic counselors (physicians and non-physicians), oncologists, surgeons, gynecologists, and trained nurses, should be authorized to provide genetic counseling as the counseling of patients is primarily focused on direct therapeutic implications and recurrence risks^[^8^]^.

Extending the patient’s overall survival and improving the quality of life (QoL) is the main objective of the management of breast cancer. Treatment strategies such as chemotherapy, targeted therapy, hormonal therapy, immunotherapy, and radiation therapy could control the disease, reduce its growth, and lengthen the survival even for incurable breast cancer. QoL can be significantly impacted by the side effects of treatment, such as exhaustion, discomfort, nausea, hair loss, weight loss, and emotional anguish. The treatment choices should be made by the requirements and preferences of individual patients, weighing the advantages against the potential disadvantages as an essential factor^[^9^]^. Advanced metastatic breast cancer has a wide range of treatment therapies and is based on various factors, such as the characteristics of tumors, including their hormone status, the extent of metastasis, prior treatment therapies, and the general health of patient. Surgical treatment for breast cancer are usually breast conservation surgeries (lumpectomy and wide local excision) and mastectomy followed by breast reconstruction surgery. Immediate breast reconstruction surgery can improve the psychosocial, sexual and physical health of the patient^[^10^]^. As patients may undergo different treatment regimens throughout time with the aim of optimal disease management and minimal adverse effects, the timing of each treatment is equally crucial. Recent advancements in genomic and molecular testing have led to the identification of particular genetic abnormalities and biomarkers in breast cancer and individualized therapeutic choices, as HER2-positive breast cancer could be treated by targeted treatments such as trastuzumab or pertuzumab, whereas hormone receptor positive could be treated by hormonal treatments such as tamoxifen or aromatase inhibitors^[^11^]^.

Palliative care comprises symptom reduction, pain management, and fulfilling the emotional, social, and spiritual needs of the patient^[^12^]^. Supportive services, such as counseling, support groups, and integrative therapies, could assist patients and their families in overcoming the struggles associated with advanced metastatic breast cancer. The involvement of patients in clinical trials could provide access to cutting-edge therapies^[^4^]^. Immunotherapies, targeted therapies, and combination therapies are novel therapeutics and are still under clinical trials to assess their efficacy and safety. Besides the treatment of patients, clinical trials could enhance cancer research and lead to effective treatment plans. Radiotherapy has a proven role in the treatment of breast cancer and is also considered immunosuppressive^[^13^]^. However, recently it has been found that radiotherapy assists the immune system in the immunogenic cell death of tumor cells. Immunotherapy is another mode of anti-cancer treatment with promising results in the treatment of breast cancer^[^14^]^. Immunotherapy evokes a tumor-specific immune response, assisting in the annihilation of tumor cells. Although advanced breast cancer is an insidious ailment, effective intervention and timely diagnosis could considerably prevent the metastasis and the consequent morbidity and mortality^[^15^]^.

For metastatic breast cancer, it is imperative to enhance access to germline genetic testing for all patients, beyond current guidelines, to ensure early identification of genetic variants and eligibility for targeted therapies and clinical trials. This necessitates an expansion of genetic counseling services, involving training more healthcare professionals to provide comprehensive counseling tailored to individual patient needs. Personalized treatment strategies based on genomic and molecular testing results should be prioritized, with healthcare providers staying updated on advancements to offer the most effective options. Integration of palliative care early in the management process is crucial for symptom management and psychosocial support. Additionally, encouraging patient participation in clinical trials is essential to provide access to innovative therapies and contribute to ongoing research efforts, ultimately improving treatment outcomes and advancing the field of breast cancer management.

## Data Availability

Data availability is not applicable to this article as no new data were created or analyzed in this study.
